# Mutagenetic study of a novel inosine monophosphate dehydrogenase from *Bacillus amyloliquefaciens* and its possible application in guanosine production

**DOI:** 10.1080/13102818.2014.901686

**Published:** 2014-05-01

**Authors:** Jian Wang, Kuifu He, Qingyang Xu, Ning Chen

**Affiliations:** ^a^ Department of Bioengineering, Jilin University, Changchun, P.R. China; ^b^ Department of Bioengineering, Tianjin University of Science & Technology, Tianjin, P.R. China

**Keywords:** *Bacillus amyloliquefaciens*, inosine monophosphate dehydrogenase, site-directed/deletion mutation, enzymatic characterisation, genetic engineering

## Abstract

In this study, the amino acid sequence of inosine monophosphate dehydrogenase (IMPDH) from a guanosine-overproducing strain *Bacillus amyloliquefaciens* TA208 was found to be highly conserved comparing to its analogue in *B. amyloliquefaciens* FZB42, only with two substitutions of serine 166 to proline and glutamic acid 481 to lysine. To speculate on the effects of these variation sites, two reverse site-directed mutants P166S and K481E, as well as one deletion mutant IMPDH^ΔCBS^, were characterised. According to the kinetic analysis of these enzymes, site-481 is a key mutation site to affect the nicotinamide adenine dinucleotide (NAD+) affinity, which accounted for the higher catalytic efficiency of IMPDH. On the contrary, mutants P166S and IMPDH^ΔCBS^ did not show better catalytic activity compared to normal IMPDH. Moreover, the overexpression of IMPDH-encoding gene *guaB* in *B. amyloliquefaciens* TA208 could improve the total production of guanosine up to 13.5 g L^−1^, which was 20.02% higher than that of the original strain.

## Introduction

Guanosine has a wide range of applications in the field of medicine and food industry. Traditionally, *Bacillus* and *Corynebacterium ammoniagenes* strains derived from mutagenic procedures were used for producing guanosine and other nucleosides and nucleotides. Recently, through genetic engineering approaches such as overexpression of key enzyme(s), optimising the export system and knockout metabolic branch pathway, *Bacillus* and wild-type *Escherichia coli* W3110 [1,2] were endowed with the capability of nucleoside accumulation. Together with co-workers, we have just recently acquired the complete genome sequence of *Bacillus amyloliquefaciens* TA208 (*Ba*-TA208), an important industrial strain used for guanosine production.[3] The overproduction of guanosine mainly depends on the activity of inosine monophosphate dehydrogenase (IMPDH, E.C. 1.1.1.205); the rate-limiting enzyme catalyses a transformation from inosine monophosphate (IMP) to xanthine monophosphate in the biosynthesis of guanosine. IMPDH from *Ba*-TA208 was intensively studied in this work to determine its genetic and enzymatic characteristics for the possible improvement of guanosine production.

Unlike *Ba-*TA208, *B*. *amyloliquefaciens* FZB42 (*Ba-*FZB42) is a typical *Bacillus* strain that could promote the growth of plants without accumulation of guanosine.[4] The sequence alignment of the *guaB* gene from *Ba-*TA208 and *Ba-*FZB42 revealed two nucleic acid variations at position 496 (T to C variation) and position 1441 (A to G variation), which leads to the replacement of two amino acids in *Ba-*FZB42 with serine 166 replaced by proline and glutamic acid 481 replaced by lysine. IMPDH has an unusual structure with a subdomain of c. 120 residues, which is composed of two repeats of a sequence known as the cystathionine β-synthase (CBS) domain.[5] To evaluate the possible effect of individual variation on the activity of IMPDH, two reverse site-directed mutants and one deletion mutant of *Ba-*TA208 *guaB* ^ΔCBS^ (serine 166 mutation located in the CBS subdomain of IMPDH) were constructed based on IMPDH sequence of *Ba-*FZB42. Kinetic analysis of purified enzymes was performed. In addition, IMPDH was overexpressed in *Ba-*TA208 to enhance the production of guanosine.

## Materials and methods

### Bacterial strains, plasmids, transformation

The bacterial strains, plasmids and primers used in this study are listed in Table 1. Plasmid DNA was transformed into *E. coli* and *B. amyloliquefaciens* by electroporation.[6] For the selection of transformants, selective antibiotic (*E. coli*, 100 mg L^−1^ ampicillin; *B. amyloliquefaciens*, 10 mg L^−1^ kanamycin) was added to the Luria-Bertani (LB) plate. Incubation temperature was 37 °C.

**Table 1.** Strains, plasmids and primers used in this study.

	Genetic characteristics	Source
Strains		
*E. coli* BL21 (DE3)	F^−^, *hsdS*_B_ (*r*_B_^−^*m*_B_^−^), *gal* (λ* cI*857, *ind*1, *Sam*7, *nin*5, *lac*UV5-T7*gene*1), *dcm* (DE3)	Lab stock
*B. amyloliquefaciens* TA208	Ade^−^, 8-AG^r^, *GuaC^−^*, 6-MP^r^ (overproducing guanosine)	Lab stock
Plasmids		
PET-His	Expression vector, 2.9 kb, Amp^r^ (T7-polymerase)	Lab stock
PDG148	*E. coli*–*Bacillus* shuttle plasmid, 8.3 kb, Amp^r^, Kana^r^	Lab stock
Primers		
P1	GCGA*GGATCC*TGGGAAAGTAAATTTTCAAAAG	This study
P2	AGTG*GCTAGC*ATTATGAGATTGTGTAGTTTGGTG	This study
P3	TGTGGTTCCCACAGAAGCCGTAA	This study
P4	GGCTTCTGTGGGAACCACATTAG	This study
P5	GTTTGGTGATTCCTTCGTGATTTG	This study
P6	TCACGAAGGAATCACCAAACTACAC	This study
P7	TCCGTGTTCATCTTTGCCCCGTTCAGAACGTTTGACTT	This study
P8	TCAAACGTTCTGAACGGGGCAAAGATGAACACGGACGC	This study
GS	TGA*GTCGAC*CGTTTTCTAGTTGATAATCT	This study
GA	TGA*GCATGC*TTCAACTTCAAAACACAACA	This study

Note: Ade^−^, adenosine monophosphate-auxotrophic; 8-AG^r^, 8-azaguanine resistance; *GuaC^−^*, deficient in GMP reducase; 6-MP^r^, 6-mercaptopurine resistance; Amp, ampicillin; Kana, kanamycin.

### Construction of expression plasmids

The DNA mutagenesis segments (P166S, K481E, *guaB* ^ΔCBS^) were constructed using *Ba-*TA208 chromosomal DNA as the template via overlapping polymerase chain reaction (PCR). The DNA fragments around the mutation sites were amplified using primer pairs P1/P3-P2/P4, P1/P5-P2/P6 and P1/P7-P2/P8. Then, the corresponding two fragments were subjected to crossover PCR using the primer pair P1/P2. Each obtained *guaB* gene with designed mutation site was cloned into PET-His expression vector via *Bam*HI and *Nhe*I restriction sites, and then transformed into *E. coli* BL21(DE3) for enzyme expression. For overexpression of homogenous IMPDH, the *guaB* gene was amplified and cloned into PDG148, and then the expression plasmid was electroporated into *Ba-*TA208.

### Expression, purification, activity assay and kinetic study of enzymes

The expression, purification, SDS-PAGE analysis and protein concentration assay of enzyme were described previously.[7] The standard reaction mixture contained appropriate recombinant enzyme, 0.2 mmol L^−1^ IMP, 5 mmol L^−1^ NAD^+^ and enzymatic reaction buffer (50 mmol L^−1^ sodium phosphate buffer, pH 8.0) in a total volume of 500 μL. The reaction was initiated by the addition of NAD^+^ and incubated at 40 °C for 2 min. Then, the mixture was cooled on ice and centrifuged at 10,000 *g* and 4 °C for 30 min. The enzymatic activity of IMPDH was measured by monitoring the formation of NADH via optical density measurement at 340 nm. One unit of IMPDH activity was defined as the amount of enzyme that causes an increase of 0.001 in absorbance at 340 nm min^−1^.

Enzyme and substrate were incubated at various temperatures from 0 to 75 °C for 2 min in 50 mmol L^−1^ sodium phosphate buffer (pH = 8.0), and the residual enzymatic activity was measured. The highest enzymatic activity measured was set as 100%. A total reaction volume of 500 μL contained appropriate recombinant enzyme, 12 mmol L^−1^ NAD^+^ and various concentrations of IMP (0.05, 0.1, 0.2, 0.3, 0.4, 0.6 and 0.8 mmol L^−1^) in the enzymatic reaction buffer. The effect of pH on the activity of IMPDHs was determined with the following buffers: 50 mmol L^−1^ citric acid buffer (pH 4–6), 50 mmol L^−1^ sodium phosphate buffer (pH 6–8) and 50 mmol L^−1^ glycine–NaOH buffer (pH 9–11) (the pH of all buffers was adjusted at 40 °C). The highest enzymatic activity measured was set as 100%. A total reaction volume of 500 μL contained appropriate recombinant enzyme, 0.2 mmol L^−1^ IMP, 5 mmol L^−1^ NAD^+^ and various concentrations of guanosine monophosphate (GMP) (0.1, 0.2, 0.4, 0.6, 0.8 and 1 mmol L^−1^) in the enzymatic reaction buffer. The highest activity measured was set as 100%. Data are represented as Mean ± SD (*n* = 3).

### Media and analytical methods

For fermentation production of guanosine, the seed medium contained 20 g L^−1^ of glucose, 5 g L^−1^ of monosodium glutamate, 10 g L^−1^ of yeast extract, 30 mL L^−1^ of corn steep liquor, 20 mL L^−1^ of soybean hydrolysates and 2.5 g L^−1^ of NaCl; the fermentation medium contained 80 g L^−1^ of glucose, 15 g L^−1^ of monosodium glutamate, 10 g L^−1^ of yeast extract, 20 mL L^−1^ of soybean hydrolysates, 15 mL L^−1^ of corn steep liquor, 15 g L^−1^ of (NH_4_)_2_SO_4_, 4 g L^−1^ of MgSO_4_·7H_2_O, 0.01 g L^−1^ of FeSO_4_·7H_2_O, 0.01 g L^−1^ MnSO_4_·H_2_O, 2 g L^−1^ K_2_HPO_4_·3H_2_O and 2 g L^−1^ CaCl_2_; 10 mg L^−1^ kanamycin was preferentially added with the strain requirement. Glucose in fermentation medium was sterilised separately. The pH was adjusted to 6.7 with NaOH before sterilisation. For guanosine fermentations, 3 mL of the obtained seed culture grown to an OD_600_ = 8 at 36 °C was inoculated into a 500-mL shake flask containing 27 mL of fermentation medium and incubated in a rotary shaker at 36 °C. Because the complex medium was used in the fermentation process, isopropyl β-D-thiogalactoside (IPTG) was not added for induction.

Guanosine was detected using an high-pressure liquid chromatography (HPLC) system equipped with a Zorbax SB-C8 column (Kromasil, C18-5 μm, 250 × 4.6 mm) at 30 °C and a UV detector at 254 nm. The mobile phase was 10% acetonitrile (V/V) and the flow rate was 1 mL min^−1^. All experiments were performed in triplicate, and the average value of these experimental data was calculated and applied.

## Results and discussion

### Optimal conditions for IMPDH and its mutant forms

**Figure 1.**
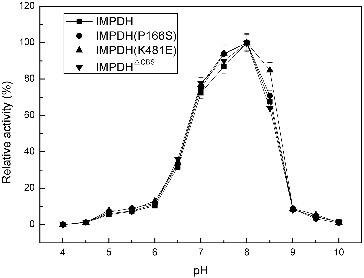
Effect of pH on the activity of IMPDHs.

**Figure 2.**
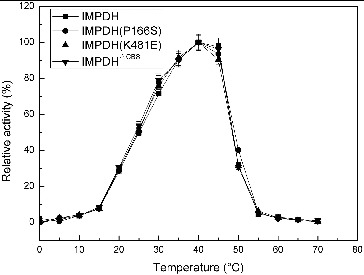
Effect of temperature on the activity of IMPDHs.

To evaluate the possible effect of individual variation on the activity of IMPDH, two reverse site-directed mutants and one deletion mutant of *Ba-*TA208 *guaB* ^ΔCBS^ were constructed based on the IMPDH sequence of *Ba-*FZB42. IMPDH and its three mutant forms, IMPDH (P166S), IMPDH^ΔCBS^ and IMPDH (K481E), exhibited similar preference for optimal pH and temperature (Figures 1 and 2). The pH curves (Figure 1) and temperature curves (Figure 2) of four IMPDHs presented similar properties as described before,[8] and showed a narrow optimal pH ranging from 7.7 to 8.0; while the optimal temperature of 40 °C is much higher than that of the IMPDH from *B. subtilis* (25 °C).

### Kinetic studies of IMPDH and its mutants

As summarised in Tables 2 and  3, the kinetic parameters of IMPDH (P166S) and IMPDH^ΔCBS^ were roughly similar to that of IMPDH; while IMPDH (K481E) exhibited varied kinetic properties compared to IMPDH. Both *k*_cat_^NAD+^ and *K*_m_^NAD+^ of IMPDH were higher than those of IMPDH (K481E); and the *k*_cat_^NAD+^/*K*_m_^NAD+^ of IMPDH was approximately 304% higher than that of IMPDH (K481E) at the same conditions. However, when IMP was used as the substrate, the kinetic constant of each of the four IMPDHs was almost the same. Thus, Ser481 was a key site to affect the substrate affinity of NAD^+^, which could account for the higher IMPDH activity in *Ba-*TA208. Above all, the improved IMPDH catalytic efficiency might be one of the reasons why *Ba-*TA208 could overproduce guanosine. It was reported that the CBS subdomain of IMPDH plays an important role in the regulation of purine nucleotide metabolism, and knockout of CBS subdomain in *E*. *coli* could strongly disturb the nucleotide pool and reduce the IMPDH activity.[8] But our result was in agreement with Nimmesgern et al.,[9] Gan et al.[10] and Zhou et al.,[11] who suggest that deletion of the CBS subdomain is unlikely to render IMPDH less catalytically active.

**Table 2.** Kinetic studies of different IMPDHs with IMP as substrate.

Enzyme	*V*_max_ (U)	*k*_cat_ (s^−1^)	*K*_m_ (μM)	*k*_cat_/*K*_m_ (s^−1^ · M^−1^)
IMPDH	1.1 × 10^2^	5.1	1.2 × 10^2^	4.2 × 10^4^
IMPDH (P166S)	75	5.0	1.2 × 10^2^	4.3 × 10^4^
IMPDH (K481E)	89	5.0	1.2 × 10^2^	4.2 × 10^4^
IMPDH^ΔCBS^	1.1 × 10^2^	5.0	1.3 × 10^2^	4.0 × 10^4^

**Table 3.** Kinetic studies of different IMPDHs with NAD^+^ as substrate.

Enzyme	*V*_max_ (U)	*k*_cat_ (s^−1^)	*K*_m_ (μM)	*k*_cat_/*K*_m_ (s^−1^ · M^−1^)
IMPDH	1.5 × 10^2^	15	3.1 × 10^3^	4.9 × 10^3^
IMPDH (P166S)	1.4 × 10^2^	15	3.2 × 10^3^	4.6 × 10^3^
IMPDH (K481E)	42	4	2.7 × 10^3^	1.6 × 10^3^
IMPDH ^ΔCBS^	1.5 × 10^2^	16	3.1 × 10^3^	5.2 × 10^3^

### Inhibition test of IMPDH and its mutants by GMP

**Figure 3.**
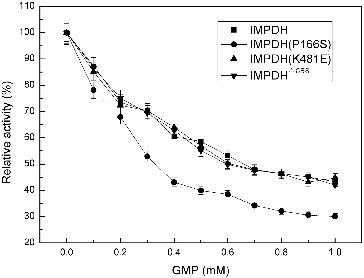
Effect of GMP on the activity of IMPDHs.

Generally, IMPDH was strongly inhibited by the final product GMP.[8] To examine the sensitivity of IMPDH from *Ba-*TA208 towards GMP *in vitro*, different concentrations of GMP (from 0 to 1.0 mmol/L) were added into the standard reaction mixture. The results (Figure 3) indicated that all four IMPDHs were partially inhibited by GMP, and IMPDH (P166S) was the most sensitive one to GMP. However, even with the highest concentration of 1.0 mmol L^−1^ GMP in the reaction mixture, IMPDH retained 40%–50% activity. These results clearly demonstrated that the IMPDH was not desensitised to GMP inhibition.

### Effect of *guaB* overexpression on production of guanosine

**Figure 4.**
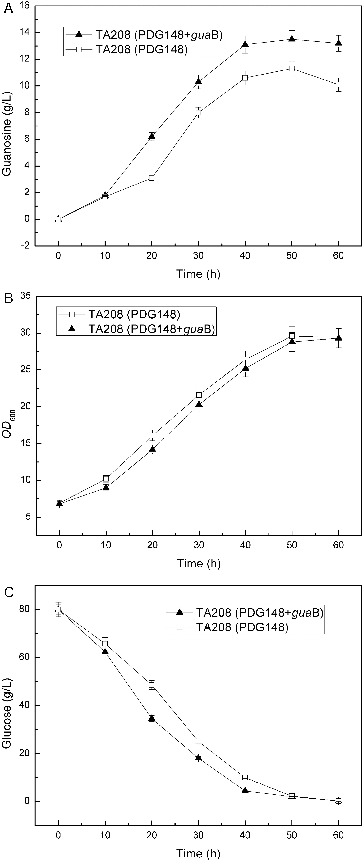
Effect of overexpression of *guaB* gene on guanosine accumulation (A), growth (B) and glucose consumption (C).

There was an augment that the higher activity of IMPDH may lead the carbon flux towards GMP production, a primary by-product that reduces the yield of guanosine and complicates the final separation process. To investigate the possible relevance between IMPDH and production of guanosine in *Ba-*TA208, the *guaB* gene was cloned into PDG148, an expression vector for systematic protein overproduction in *Bacillus*, and transformed back into *Ba-*TA208. Comparing to the reference with an empty vector only, the overexpression of *guaB* in *Ba-*TA208 resulted in a steady cell growth rate and 20.02% higher production of guanosine (Figure 4). This result clearly indicated that IMPDH is closely joined with the biosynthesis of guanosine, and the overexpression of IMPDH in host cells could be useful to improve the guanosine production. Similarly, overexpression of the IMPDH of guanosine-producing *B*. *subtilis* NA7821 in an inosine-producing *B*. *subtilis* NA6128 resulted in an increase of IMPDH activity and a higher guanosine productivity accompanied by a decreased accumulation of inosine.[12]

Currently, the industrial guanosine-producing strains are obtained through random mutagenesis, which depends on a chance to encounter desired mutants among resulting colonies which inevitably accumulate numerous unidentifiable and unwanted mutations. Therefore, for the next generation of microbial breeding, efficient and selective gene-targeted mutagenesis is required.[1] The present study could result in the development of effective engineering of the guanosine-producing strain.

## Conclusions

Since the overproduction of guanosine mainly depends on the activity of IMPDH, IMPDH from *Ba*-TA208 was intensively studied in this work. To evaluate the possible effects of individual variation on the activity of IMPDH, two reverse site-directed mutants and one deletion mutant of *Ba-*TA208 *guaB* ^ΔCBS^ were constructed based on the IMPDH sequence of *Ba-*FZB42. Kinetics analysis of purified enzymes indicated that the substitution of glutamic acid to lysine at 481 of IMPDH could evidently reduce its affinity with substrate NAD^+^, thus impairing its enzymatic activity and subsequent production of guanosine. In addition, our results also suggested that the overexpressing of IMPDH in *Ba-*TA208 could enhance the production of guanosine with no significant effect on the growth rate. Thus, our work revealed the practical potential of this novel IMPDH from *Ba-*TA208 for further improvement of guanosine production. The study of this novel IMPDH from *B. amyloliquefaciens* described here should also be useful for constructing other strains that produce and export high levels of purine-related products.
